# Safety of Drinking Water from Primary Water Sources and Implications for the General Public in Uganda

**DOI:** 10.1155/2019/7813962

**Published:** 2019-03-25

**Authors:** Keneth Iceland Kasozi, Sarah Namubiru, Roland Kamugisha, Ejike Daniel Eze, Dickson Stuart Tayebwa, Fred Ssempijja, Alfred Omachonu Okpanachi, Hellen Wambui Kinyi, Jovile Kasande Atusiimirwe, Joy Suubo, Edgar Mario Fernandez, Nathan Nshakira, Andrew Tamale

**Affiliations:** ^1^Department of Physiology, Faculty of Biomedical Sciences, Kampala International University Western Campus, Box 71, Bushenyi, Uganda; ^2^College of Veterinary Medicine Animal Resources and Biosecurity, Makerere University, Box 7062, Kampala, Uganda; ^3^School of Pharmacy, Kampala International University Western Campus, Box 71, Bushenyi, Uganda; ^4^Department of Anatomy, Faculty of Biomedical Sciences, Kampala International University Western Campus, Box 71, Bushenyi, Uganda; ^5^Department of Biochemistry, Faculty of Biomedical Sciences, Kampala International University Western Campus, Box 71, Bushenyi, Uganda; ^6^Department of Biochemistry, Faculty of Biomedical Sciences, School of Medicine, Kabale University, Uganda; ^7^Department of Public Health, Faculty of General Medicine and Public Health, School of Medicine, Kabale University, Uganda; ^8^Department of Public Health, School of Allied Health and Medicine, Kampala International University Western Campus, Box 71, Bushenyi, Uganda

## Abstract

**Background:**

There is scarcity of information about the quality and safety of drinking water in Africa. Without such vital information, sustainable development goal number 6 which promotes availability and sustainable management of water and sanitation remains elusive especially in developing countries. The study aimed at determining concentrations of inorganic compounds, estimated daily intake (EDI), target hazard quotient (THQ), hazard index (HI), incremental lifetime cancer risk (ILCR), and identify safe drinking water source sources in Southwestern Uganda.

**Methods:**

This was an observational study in which 40 drinking water samples were collected from georeferenced boreholes, springs, open wells, bottled, and taps within Bushenyi district of Southwestern Uganda. Water samples were analyzed for copper (Cu), iron (Fe), zinc (Zn), lead (Pb), cadmium (Cd), and chromium (Cr) levels using atomic absorption spectrometry (AAS). Water safety measures (EDI, HI, and ILCR) were established for each water source and compared with local and international water permissible standards for each analyte. A spatial map was drawn using qGIS®, and analysis of quantitative data was done using MS Excel 2013 at 95% significance.

**Results:**

Heavy metals were present in the following order: 11.276 ppm > 4.4623 ppm > 0.81 ppm > 0.612 ppm > 0.161 ppm for Fe, Zn, Pb, Cu, and Cd, respectively, while Cr was not detected. Fe was the primary water heavy metal in the order of open well > borehole > tap > spring > bottled water. This was followed by Zn levels in the order of tap > bottled > spring > borehole > open well. All compounds were within international water safety standards except Pb. Hence, there is need for the government of Uganda to establish water filtration systems, particularly for Pb to improve the quality of water for the general public. The EDI was similar (*P* > 0.05) for water consumed from spring, bottled, and tap sources for Fe and Zn levels. Similarly, no differences were found in the EDI for children and adults (*P* > 0.05). Furthermore, the HI showed an absence of noncarcinogenic risk associated (HI < 1), although the ILCR was higher in adults than children (*P* < 0.05) due to high Cd concentrations.

**Conclusion:**

The current identified Fe is a major heavy metal in drinking water of Uganda, and boreholes were the major safest sources of drinking water identified in this study.

## 1. Background

Access to safe drinking water is considered a universal human right by the United Nations convention [1, 2]; however, this human right remains a dream for several developing countries in Asia, South America, and Africa. Previous studies have placed a lot of attention on the microbial load with a focus on infectious diseases [3, 4], while little information is available regarding the heavy metal concentrations in drinking water of Uganda [5, 6]. The lack of proper water treatment and increased agrochemical use and industrial growth suggest that water contamination is ongoing and is a threat to public health [6, 7]; therefore, baseline data on the safety of drinking water in Uganda could inform mitigation measures to ensure access to safe water in Uganda [8]. This would inevitably help Uganda remain on the path for the attainment of Sustainable Developmental Goal (SDG) number six which promotes access to safe drinking water [9].

In Kampala, the capital city of Uganda, previous studies identified high levels of heavy metals such as lead (Pb), zinc (Zn), iron (Fe), copper (Cu), cadmium (Cd), and chromium (Cr) were detected in drinking water [10, 11]. Similarly, those compounds have also been isolated in natural water reservoirs including lakes [12, 13], wetlands [12], fish [14, 15], and beef and milk [16]. Such contaminations pose a public health threat to Ugandans. Pb is present in petrol, paints, and water pipes and in soils within our environment [17]. It is medically used in X-ray shielding; however, information on effective waste management of Pb in Africa is limited to date, although it continues to be a global water contaminant [18–20]. In Zambia, Pb toxicity has been reported in children, and this has been associated with anemia, abdominal pains, limb pains, memory problems, headaches, weakness in hands and feet, and seizures or convulsions in humans [21, 22]; however, evidence on its carcinogenic effects in humans is limited to date [23, 24]. Cd has an established carcinogenic potential in humans, and it is often deposited in tissues in bone tissue as it substitutes for calcium to cause toxicity [25]. Cd primarily arises from soil sediments, batteries, and plastics which eventually contaminate water within the ecosystem [26]. In rats, Cd has been associated with development of respiratory tumors, and in humans, it has been linked to the prostate, kidney, and lung cancers [26–28]. Cd levels once at high levels in the environment have been shown to contaminate drinking water through increased ionic leakage into the water table [10, 29, 30] Zn is important in neurotransmission as a micronutrient since high levels can suppress Fe and Cu absorption in the gastrointestinal tract and has strong anticancer effects at high concentrations [31, 32]. Fe and Cu are micronutrients important in hemoglobin and neurofunction, respectively [16]. Cr is present in the environment in rocks, plants, and soils and is a known human carcinogen associated with stomach cancers [16, 33, 34]. Currently, Cr is used as a metal coating, pigments for paints, cement, paper, rubber, and floor coverings and commonly used as a wood preservative [35]. In water, Cr is effectively removed by coagulation-filtration on a large scale, while adsorptive filtration and ion exchange are appropriate for large-and small-scale applications [36].

Several human-based practices that include pesticide application and industrialization are associated with soil and plant contamination. Subsequently, after the heavy rains, the runoff water carries the pollutants to water reservoirs from which humans and animals consume the contaminated water [37–39]. Heavy metals subsequently bioaccumulate in the bodies of animals and humans predisposing them to cancer and other public health risks following oral ingestion [7, 40]. As a short-term solution, the installation of filters against major heavy metals would improve the safety of the consumer [29, 41]. In Uganda, major drinking water sources are borehole water, bottled water, open well water, spring water, and tap water [8, 10, 42]. Therefore, for Uganda to maintain her path to attain Goal 6 of the SDGs, there was a need to ensure that drinking water in rural communities met international standards. The scarcity of information regarding the levels of heavy metals in drinking water from major water sources in Uganda indicated a knowledge gap that necessitates the concerned bodies such as the Uganda National Water and Sewerage Cooperation (UNWSC) and the Uganda National Bureau of Standards (UNBS) to take action [9, 20]. Lest Ugandans are exposed to pollutants which expose the people in communities to various health risks including cancer [16, 43]. Therefore, the objective of this study was to measure the concentrations of Cu, Fe, Zn, Pb, Cd, and Cr, estimate the daily intake, estimate the presence of major cancer and noncancer health risks, and identify safe water sources for the people in Southwestern Uganda.

## 2. Methods

### 2.1. Study Design

**Figure 1:**
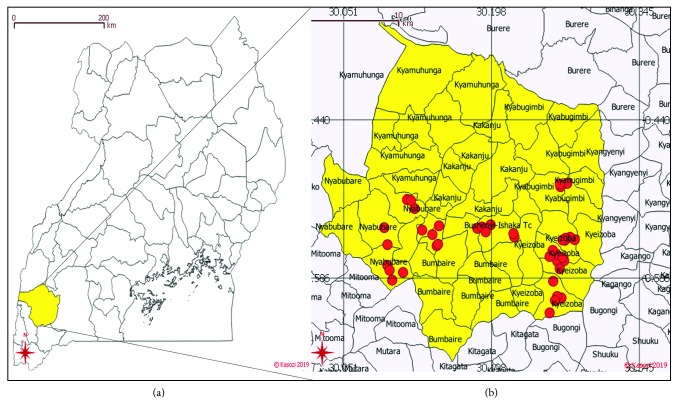
Map showing water sources within Bushenyi district of Uganda: (a) map of Uganda showing Bushenyi district in yellow; (b) map of Bushenyi showing survey points.

This was a cross-sectional study conducted in Bushenyi district of Southwestern Uganda in July 2017. Bushenyi district lies 330.4 km from Kampala Capital city of Uganda by road. Bushenyi is bordered by Rubirizi, Buhweju, Sheema, Mitooma, and Rukungiri districts to the northwest, northeast, east, south, and west, respectively. Ishaka is its largest town which is located 75 km by road from Mbarara district which is the largest city in the region. The district coordinates are 00 32S, 30 11E. In our previous study [16], milk and beef from Bushenyi district were contaminated with heavy metals; therefore, we performed this study as a follow-up to understand the source of the contamination. The simple random sampling technique was used from which 4 subcounties in Bushenyi district were included: Ishaka-Bushenyi Municipality, Kyeizoba, Kyabugimbi, and Kigoma-Nkanga. A total of 40 drinking water samples were randomly collected. In each major trading center, 2 samples, each of 50 ml were collected into 50 ml falcon tubes using aseptic techniques from the borehole water (BHW), commercial bottled water (BotW), open well (OW), spring water (SW), and tap water (TW). The water was collected into sterile falcon tubes carefully avoiding any contamination. Georeferencing of the water samples was performed and recorded with an acceptable accuracy of 3 m using a GPS Garmin from the Uganda Government and recorded in MS Excel, and mapping was conducted using qGIS® 3.03 as shown in Figure 1. The samples were coded and taken to the laboratory for Fe, Cu, Zn, Pb, Cd, and Cr analysis as previously described [16].

### 2.2. Laboratory Analysis

Usually nitric acid is used for digestion of solid samples and waste water. Since this was drinking water, the use of nitric acid was to serve this particular purpose. It is the reason the statement is brought to the attention of the reader. Distilled water was used to prepare solutions and for dilution purposes. All glassware were washed and dried in the oven at 105°C. Bottles for collecting water samples were cleaned by soaking in dilute nitric acid (10%) and rinsed several times with distilled water prior to sample collection [10]. Wet digestion of the samples was subsequently done using 30 ml of nitric acid at 150°C for 45 minutes. The solution was left to evaporate up to 10 ml, and 2 ml of hydrogen peroxide was added followed by deionized water up to 30 ml. The solution was then transferred to a plastic bottle ready for analysis. The water sample solutions were analyzed with an atomic absorption spectrophotometer (AAS) (PerkinElmer 2380), which had detection limits for Pb at 0.01 ppm while for Cu, Fe, and Cr at 0.001 ppm were used as previously described [16]. Linear equations for each metal were generated in the form *y* = *mx*; where *y* = absorbance, *m* = gradient, and *x* = concentration for each compound using standard working standard stock solutions for the heavy metals acquired from E. Merck, D-6100, Darmstadt, FR, Germany, as previously described [16].

  For Fe: *y* = 0.0841*x*, *R*^2^ = 0.8678
  For Cu: *y* = 0.184*x*, *R*^2^ = 0.8748
  For Zn: *y* = 0.299*x*, *R*^2^ = 0.9837
  For Pb: *y* = 0.0296, *R*^2^ = 0.8637
  For Cd: *y* = 0.1025*x*, *R*^2^ = 0.9552

These equations were used to determine the concentrations of the compounds in each sample as previously described [16].

### 2.3. Assessment of Water Safety against International Reference Standards

The data point generated was compared with those from Uganda National Bureau of Standards (UNBS), the United States Environmental Protection Agency (US-EPA), European Union (EU), and the World Health Organization (WHO) using the table drawn from Bamuwamye et al. [10].

### 2.4. Determination of the Estimated Daily Intake

This was modelled using recent Ugandan projections [10] and was calculated using the equation:

(1) $$\begin{array}{c} \text{EDI}=\frac{\left(C\times \text{IR}\right)}{\text{BW}}, \end{array}$$

where *C* = concentration of the metal (mg/kg), IR = ingestion rate for water, and BW = body weight. In children and adults, IRs were 1 L/day and 2 L/day while the body weight for was 15 kg and 70 kg, respectively [10].

### 2.5. Determination of the Noncancer Risk Associated with Drinking Water in Uganda

The target hazard quotient (THQ) was used to generate the hazard index (HI) to determine presence of noncarcinogenic health effects following ingestion of the sampled water. The THQ was determined for Pb, Zn, Cd, Cu, and Fe (US EPA) [44] using the following equation:

(2) $$\begin{array}{c} \text{THQ}=\frac{\text{CDI}}{\text{RfD}}, \end{array}$$

where CDI = exposure dose obtained and RfD is the oral reference dose of the contaminant. The RfD is an estimation of the maximum permissible risk on human population through daily exposure.

(3) $$\begin{array}{c} \text{CDI}=\frac{\left(\text{EDI}\times \text{EFr}\times \text{EDtot}\right)}{\text{AT}}, \end{array}$$

where EDI is the estimated daily intake of a metal via ingestion of specific route; EFr is the exposure frequency (365 days/year); EDtot is the exposure duration (i.e., 6 years for children and 30 years for adults); and AT  is the period of exposure for noncarcinogenic effects (it is equal to EFr × EDtot, i.e., 2190 days in children and 10950 days in adults). Furthermore, the reference dose (RfD) for each hazard was obtained from the US EPA [44], i.e., 0.004 ppm, 0.3 ppm, 0.001 ppm, 0.04 ppm, and 0.7 for Pb, Zn, Cd, Cu, and Fe, respectively. Exposure to multiple contaminants results in additive and interactive effects; thus, the hazard index (HI = ∑THQ) was used as an indication of risk.

### 2.6. Determination of the Incremental Lifetime Cancer Risk Associated with Drinking Water amongst Ugandans

Following chronic exposure to inorganic pollutants in drinking water, the incremental lifetime cancer risk (ILCR) was used to model the cancer risk in the Ugandan population. This was estimated using the following equation:

(4) $$\begin{array}{c} \text{ILCR}=\text{CDI}\times \text{CSF}, \end{array}$$

where CDI is the chronic daily intake of a particular metal and this was estimated over the 70-year lifespan for Ugandans (i.e., AT = 70 yrs × *x*365 days = 25550 days) [16, 45]. In addition, the cancer slope factor (CSF) for Cd that was used was 6.3 [23, 25].

### 2.7. Spatial Map on Safe Water Sources in Study Area

Information acquired from the GPS readings was exported to qGIS® version 3.03 Cirona onto an administrative file for Uganda. A sentinel-2 satellite image number L1C_T3MRV_AO07540_20180816T082305 was acquired from the United States Geographical Surveys (USGS) system to show vegetation cover in the study area.

### 2.8. Statistical Analysis

Data were entered and analyzed in MS Excel 2013 version after normality testing, after which parametric tests were conducted. Descriptive statistics were conducted and information was presented as mean ± SEM from which a one-way ANOVA was conducted, and significant differences were reported when *P* < 0.05. Information on safety was done using a one-sample *t*-test, and mean differences were used to define “high” and “low” after subtracting the sample mean from the hypothetical mean. These were used to define safety of drinking water at 95% significance. Furthermore, the EDI for children and adults for each metal was presented as mean ± SEM, and a two-sample *t*-test was conducted to determine differences in concentrations ingested and significance reported when *P* < 0.05. The HI was calculated to assess the presence of threat, i.e., HI > 1 as an indicative of a threat [16]. Also, significant differences in the THQs for children and adults were determined at 95% significance. Finally, ILCR was presented descriptively, and a two-sample *t*-test for children and adults was conducted at 95% significance. ILCR greater than 1 × 10^−4^ was an indicative of a cancer threat and presented with superscripts [16, 25].

## 3. Result

### 3.1. Levels of Heavy Metals from Different Water Sources in the Study Area

The study showed that Fe is the major water pollutant in the order of open well > borehole > tap > spring > bottled water. This was followed by high Zn levels in the order of tap > bottled > spring > borehole > open well. Cu and Pb concentrations were relatively comparable where Cu was found to be high in the order of tap > borehole > spring > open well > bottled. Furthermore Pb concentrations were found to be in the order of open well > borehole > tap > bottled > spring. Finally, Cd levels were found to be the lowest in all water samples; however, these were highest in the order of tap > open well, bottled water > spring > borehole. Cr was not detected in all water samples as shown in Table 1.

### 3.2. Safety of Drinking Water in Southwestern Uganda

Concentrations of Fe, Zn, and Cu were permissible by local and international regulatory agencies, while levels of Pb were found to be unacceptable in all water samples except borehole water using UNBS, EU, and WHO cutoff limits. The study also showed that Cd was acceptable using US-EPA and EU cutoffs as shown in Table 2. Findings in the study show that boreholes were the safest source for drinking water in Uganda.

### 3.3. Levels of Heavy Metals Consumed Daily in Drinking Water by Ugandans in Study Area

Ingestion of Zn and Cd from borehole drinking water was found to be significantly different amongst children and adults (*P* < 0.05). Also, bottled water ingestion of Pb and Cd was different amongst children and adults (*P* < 0.05). In open well drinking water, daily ingestion of Cu, Pb, and Cd were found to be different amongst children and adults, while significant differences in spring and tap water were only limited to Pb and Cd (*P* < 0.05) as shown in Table 3.

**Figure 2:**
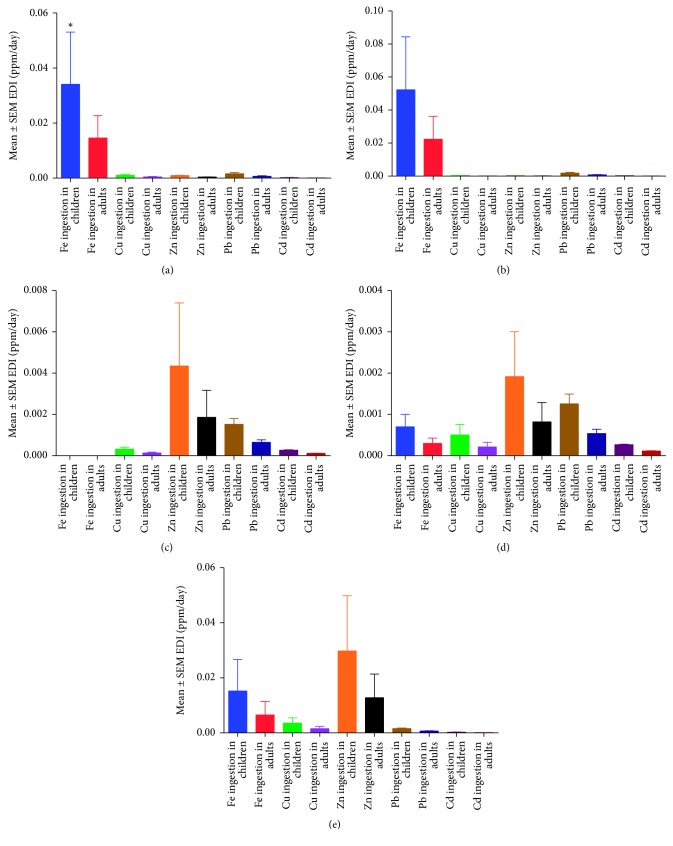
Modelled estimation of inorganic compound ingestion amongst children and adults in Uganda. (a) Borehole water. (b) Open well water. (c) Bottled water. (d) Spring water. (e) Tap water.

The study also showed that Fe ingestion was highest in children than adults from both borehole and open wells. Fe ingestion was in the order of open well > borehole > tap > spring > bottled. Zn ingestion was also found to be highest in tap > bottled > spring water as shown in Figure 2.

### 3.4. Noncancer Health Hazards Associated with Drinking Water amongst Ugandans in Study Area

The hazard index showed that all water sources were acceptable (HI < 1). The study also showed that the target hazard quotient (THQ) was significantly (*P* < 0.05) higher in children than in adults for Pb > Cd > Zn on borehole water. Open well water had significantly higher THQ in the order of Pb > Cd > Cu > Zn in children than in adults. In addition, bottled, spring, and tap water all had significantly higher THQs in children for Pb > Cd than in adults as shown in Table 4.

**Figure 3:**
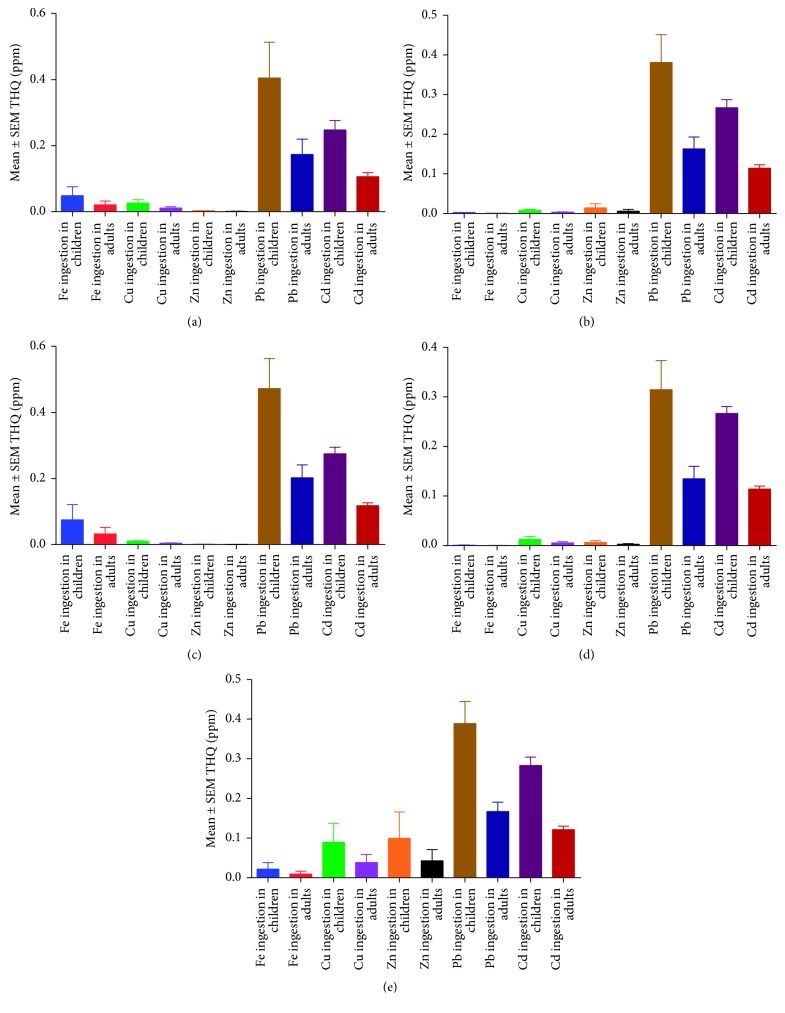
Modelled estimation of target hazard quotients (THQ) for children and adults in Uganda following ingestion of drinking water from different sources. (a) Borehole water source. (b) Open well water source. (c) Bottled water source. (d) Spring water source. (e) Tap water source.

Increased ingestion of drinking water from borehole, bottled, open well, spring, and tap water was associated with an increased threat in children than adults due to high Pb levels. This was followed by Cd showing the relevance of Pb and Cd toxicities amongst children as shown in Figure 3.

### 3.5. Incremental Lifetime Cancer Risk Associated with Drinking Water Consumed by Ugandans in Study Area

Ingestion of drinking water from all the water sources was associated with a very low threat of cancer in children while this was present amongst adults of the Ugandan population. In particular, significant differences were shown to exist in the incremental lifetime cancer risk (ILCR) in borehole, bottled, and open well drinking water in which adults had higher ILCR than children, i.e., Cd. In addition, the ILCR for spring and tap water was only significantly higher in adults than in children for Cd as shown in Table 5. The risk of cancer was highest in the order of tap > bottled > spring > borehole > open well water especially amongst adults than children. This was shown to be primarily associated with the high Cd levels in drinking water from these different sources.

### 3.6. Map Showing the Drinking Water Sources Involved in the Current Study

**Figure 4:**
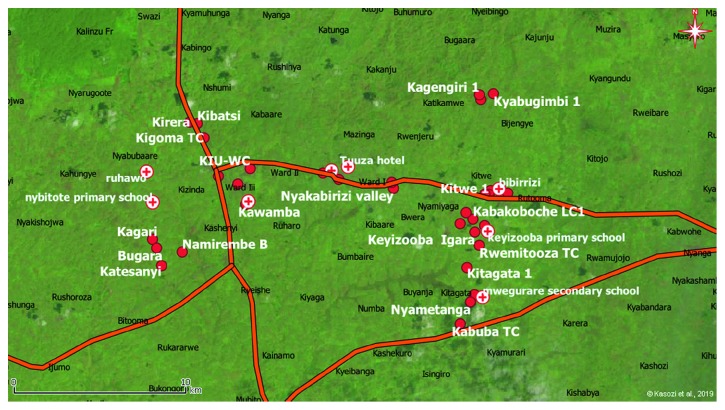
Map showing water survey points under the vegetation cover in the study area; red cross under white background = safe water; red = unsafe water. TC = trading center; LC1 = local council 1; KIU-WC = Kampala International University Western Campus. SSS = senior secondary school.

The study showed that safe drinking water was associated with boreholes, and a majority of these were located in Kitwe, Nyamiyaga, and Nyabubaare subcounties as well as Ward I of Ishaka-Bushenyi Municipality which are all agricultural areas as shown in Figure 4.

## 4. Discussion

The study showed that sampled water sources had high Fe concentrations, although Fe concentrations were highest in open water sources (Table 1). Open water sources have a high concentration of Fe in comparison to other water sources in this study possibly because the open water sources act as capture center for runoff rain water from the land which may carry Fe-rich soils [37, 38, 46]. In addition, high Zn levels in tap water (Table 1) were found to be over 100 times higher than those shown by a related study in Central Uganda [10], probably due to weaker water quality practices in rural communities of Uganda. In China, high Zn levels in drinking water have been associated with mining activities [47]; however, there was no evidence for this in Bushenyi district of Southwestern Uganda where these samples were collected showing that environmental contamination with Zn was related to the heavy pesticide usage which is common in many agricultural ecosystems [16, 48]. Furthermore, Cu concentrations were highest in tap water (Table 1) possibly due to the use of copper pipes in the transportation of water by the Uganda National Water and Sewerage Cooperation (UNWSC). Fortunately, recent developments by the UNWSC have seen an introduction of polyvinyl chloride (PVC) pipes especially in major towns within Uganda [10], although coverage is still low. At the time of the study, Bushenyi district was in the process of replacing the old pipes with PVC (personal observation in the community); however, information on the efficiency of these pipes in reducing heavy metal concentrations was limited in Uganda. Information in the study also showed no significant differences (ANOVA, *P* > 0.05) in heavy metal concentrations for Fe, Cu, Zn, Pb, and Cd showing their importance to the general public due to the threat of bioaccumulation following chronic exposure [42, 49].

Concentrations for Fe, Zn, and Cu were acceptable for human ingestion with the exception of Pb (Tables 2 and 6). In Central Uganda, high Pb toxicities in drinking water had been reported [10, 11]; however, this was the first report to document the Pb contamination in drinking water from rural communities in Southwestern Uganda. Taken together, our findings suggest that heavy metals are a major concern in both the rural and urban cities of Uganda [10, 16]. The study also showed that borehole drinking water was the safest in reference to international standards showing that water quality from other water sources was in a much worse state. The installation of appropriate filters against inorganic pollutants has been associated with increased water quality, and this could be a necessity for Uganda [41]. Currently, the quality of drinking water available to Ugandans was below international standards, and this was in agreement with previous findings in the region demonstrating the importance of our findings [8, 10, 29].

The estimated daily ingestion (EDI) of heavy metals from borehole drinking water was found to be significantly different (*P* < 0.05) for Zn and Cd amongst children > adults (Table 3). This showed that Zn levels are high in borehole drinking water of Bushenyi district which is of physiological benefit to the community [31, 32]. Furthermore, installation of Cd filters in the boreholes would make the water much safer than it currently is due to its carcinogenic risk [10, 29, 41]. In bottled water, the EDI was also highest in children than in adults for Pb > Cd (Table 3). Our findings suggest that bottled water is not necessarily recommended for children in Uganda, and this was contrary to a previous recommendation from a study conducted in central Uganda [10]. Findings in our study might be incidental or influenced by the geographical area of the study; however, a need by the UNBS in adopting practical monitoring strategies against carcinogenic compounds in drinking water has been provided in this study. Open well drinking water also showed significant variations in Cu, Pb, and Cd levels amongst children > adults (Table 3). Open drinking water was contained with heavy metals due to heavy washoff following a heavy rain down pour [37, 38, 46]. The higher ingestion of Fe in children than in adults in drinking water from boreholes and open wells (Figure 2) was related to the poor maintenance of these facilities and mineral leaching [41].

Estimation of the noncarcinogenic health effects was done by using the hazard index, and all water samples were found to be acceptable in line with international guidelines (HI < 1); however, significant differences in borehole water were in Pb > Cd > Zn higher in children > adults (Table 4). These findings are in agreement with the recent studies in Bushenyi, which have shown children to be at a higher risk than adults to heavy metal toxicity [16]. Furthermore, bottled, spring, and tap water all had significantly higher THQs in children for Pb > Cd than in adults (Table 4). This re-emphasized previous finding in central Uganda that drinking water in Uganda was contaminated with Pb [10, 11]. Access to safe drinking water is a universal human right; thus, findings in this study cannot be taken for granted by the authorities in Uganda [1, 2, 42]. This would help reduce on the health burden associated with oral ingestion of these elements, thus promoting public health [37, 43]. The study also showed that Pb and Cd toxicities are very important in children of Southwestern Uganda (Figure 3), probably due to their smaller body weights relative to adults. This is because Pb has been associated with gastrointestinal irritation which would lead to vomiting and diarrhea while Cd has been associated with brain and kidney damage in humans [24, 50].

Chronic ingestion of heavy metals at very high concentrations in drinking water would subsequently predispose the local population to toxic health effects such as cancer. In this community, the incremental lifetime cancer risk revealed the absence of a threat in children while this was present in adults due to high Pb and Cd levels (Table 5). This was important since Pb and Cd have established carcinogenic potential in humans [16,25–28]. In Bushenyi district, agricultural usage of pesticides was the major mechanism of toxicity (data not shown). This has been propagated by the poor implementation of the drug liberalization policy which has seen associated with severe drug abuse in farming communities and subsequent heavy washoff of these chemicals into major water sources of Uganda [37, 38, 46]. This has consistently led to very high heavy metal concentrations being detected in food and drinking water products of Uganda [10, 16]. The risk of cancer was found to be highest in tap water due to high Cd levels, and this was lowest in open well water. Finally, the study identified safe drinking water centers in Nyabubaare, Ward 1 of Ishaka-Bushenyi Municipality, Kitwe, and Nyamiyaga within the study area (Figures 1 and 4). Heavy metals enter the soils and groundwater, bioaccumulate in food webs, and adversely affect the ecosystem [51], demonstrating the importance of the current findings to guide policy and promote development for increased public health amongst Ugandans in rural communities.

## 5. Conclusion

Drinking water in Southwestern Uganda had high Fe and Zn concentrations. The study identified borehole water as safer source of drinking water, demonstrating a need for increased monitoring by the regulatory authorities for improved water quality. The authors recommend the installation of heavy-metal filters especially against Pb and Cd to reduce on the carcinogenic risk of drinking water in the community. For increased water quality, authorities need to continuously monitor water against major heavy metals in the study since very high levels of metals once ingested can lead to severe toxicological effects in humans.

## Figures and Tables

**Table 1:** Concentrations of different inorganic compounds from major water sources in the study area.

Water source	*N*	Mean ± SEM concentration (ppm)					
		Fe	Cu	Zn	Pb	Cd	Cr
Borehole	8	0.512 ± 0.2839	0.01586 ± 0.005784	0.0145 ± 0.001739	0.02429 ± 0.006494	0.003714 ± 0.0004206	ND
Bottled	8	0.0290^a^	0.004833 ± 0.001447	0.06523 ± 0.04579	0.02286 ± 0.004206	0.0040 ± 0.0003086	ND
Open well	7	0.7841 ± 0.4818	0.006143 ± 0.000885	0.005338 ± 0.0008608	0.02833 ± 0.005426	0.004125 ± 0.0002950	ND
Spring	9	0.0105 ± 0.0045	0.007571 ± 0.003791	0.02876 ± 0.01629	0.01889 ± 0.003514	0.0040 ± 0.0002108	ND
Tap	8	0.2282 ± 0.1719	0.05371 ± 0.02876	0.4467 ± 0.3008	0.02333 ± 0.003333	0.00425 ± 0.0003134	ND
*P* value		0.6387	0.0815	0.1283	0.7054	0.8092	

Fe = iron; Cu = copper; Zn = zinc; Pb = lead; Cd = cadmium; Cr = chromium. *N* = number of samples submitted for analysis. ND = not detected during analysis. ^a^One value included in the calculation. ANOVA conducted for all the compounds, and respective *P* values are presented. SEM = standard error mean; ppm = parts per million.

**Table 2:** Drinking water safety assessment using cutoffs from local and international regulatory agencies.

Regulatory bodies	BHW	BotW	OW	SW	TW	95% significance
	*P* values (conclusions based on mean differences)					
Fe (*N*=24)	(*N*=7)	(*N*=1)	(*N*=8)	(*N*=2)	(*N*=6)	
UNBS	0.4834 (high)	NA (low)	0.3484 (high)	0.0099 (low)	0.6934 (low)	All safe
US-EPA	0.4834 (high)	NA (low)	0.3484 (high)	0.0099 (low)	0.6934 (low)	All safe
EU	0.3139 (high)	NA (low)	0.2647 (high)	0.0151 (low)	0.8763 (high)	All safe
WHO	0.4834 (high)	NA (low)	0.3484 (high)	0.0099 (low)	0.6934 (low)	All safe

Pb (*N*=35)	(*N*=7)	(*N*=7)	(*N*=6)	(*N*=9)	(*N*=6)	
UNBS	0.0701 (high)	0.0223 (high)	0.0197 (high)	0.0353 (high)	0.0103 (high)	Accept BHW
US-EPA	0.2027 (high)	0.1109 (high)	0.0574(high)	0.3005 (high)	0.0545 (high)	All safe
EU	0.0701 (high)	0.0223 (high)	0.0197 (high)	0.0353 (high)	0.0103 (high)	Accept BHW
WHO	0.0701 (high)	0.0223 (high)	0.0197 (high)	0.0353 (high)	0.0103 (high)	Accept BHW

Zn (*N*=35)	(*N*=6)	(*N*=6)	(*N*=7)	(*N*=9)	(*N*=7)	
UNBS	<0.0001 (low)	<0.0001 (low)	<0.0001 (low)	<0.0001 (low)	<0.0001 (low)	All safe
US-EPA	<0.0001 (low)	<0.0001(low)	<0.0001 (low)	<0.0001(low)	<0.0001 (low)	All safe
EU	<0.0001 (low)	<0.0001 (low)	<0.0001 (low)	<0.0001 (low)	0.0002 (low)	All safe
WHO	<0.0001 (low)	<0.0001 (low)	<0.0001 (low)	<0.0001 (low)	0.0002 (low)	All safe

Cd (*N*=39)	(*N*=6)	(*N*=7)	(*N*=8)	(*N*=10)	(*N*=8)	
UNBS	0.1403 (high)	0.0177 (high)	0.0066 (high)	0.0011 (high)	0.0053 (high)	Accept BHW
US-EPA	0.0223 (low)	0.0117 (low)	0.0209 (low)	0.0011 (low)	0.479 (low)	All safe
EU	0.0223 (low)	0.0117 (low)	0.0209 (low)	0.0011 (low)	0.479 (low)	All safe
WHO	0.1403 (high)	0.0177 (high)	0.0066 (high)	0.0011 (high)	0.0053 (high)	Accept BHW

Cu (*N*=34)	(*N*=7)	(*N*=6)	(*N*=7)	(*N*=7)	(*N*=7)	
UNBS	<0.0001 (low)	<0.0001 (low)	<0.0001 (low)	<0.0001 (low)	<0.0001 (low)	All safe
US-EPA	<0.0001 (low)	<0.0001 (low)	<0.0001 (low)	<0.0001 (low)	<0.0001 (low)	All safe
EU	<0.0001 (low)	<0.0001 (low)	<0.0001 (low)	<0.0001 (low)	<0.0001 (low)	All safe
WHO	<0.0001 (low)	<0.0001 (low)	<0.0001 (low)	<0.0001 (low)	<0.0001 (low)	All safe

BHW = borehole water; BotW = bottled water; OW = open well; SW = spring water; TW = tap water. Fe = iron; Pb = Lead, Zn = zinc; Cd = cadmium; Cu = copper. *N* = number of samples detected by atomic absorption spectrometry (AAS). NA = not applicable since mean was not calculated. Regulatory monitoring agencies included UNBS = uganda National Bureau of Standards, US-EPA = United States Environmental Protection Agency, EU = European Union, and WHO = World Health Organization. The one-sample *t*-test conducted against respective metals with hypothetical means set by different international regulatory agencies and *P* values is included from which conclusions on safety were made.

**Table 3:** Estimated daily intake of heavy metals in drinking water amongst Ugandans.

Heavy metals in drinking water	*N*	Children	Adults	*P* values
		Mean ± SEM ppm/day		
Borehole water				
Fe	7	0.03413 ± 0.01893	0.01463 ± 0.008111	0.3708
Cu	7	0.001057 ± 0.0003856	0.0004531 ± 0.0001652	0.1872
Zn	7	0.0009667 ± 0.0001159	0.0004143 ± 0.0000497	0.002259
Pb	7	0.0016190 ± 0.0004330	0.0006939 ± 0.0001856	0.08453
Cd	7	0.0002476 ± 0.0000280	0.0001061 ± 0.0000120	0.001598

Bottled water				
Fe	1	NC	NC	NC
Cu	6	3.221*E* − 04 ± 9.651*E* − 05	1.381*E* − 04 ± 4.137*E* − 05	0.4137
Zn	7	4.349*E* − 03 ± 3.052*E* − 03	1.864*E* − 03 ± 1.308*E* − 03	0.4754
Pb	7	1.524*E* − 03 ± 2.804*E* − 04	6.530*E* − 04 ± 1.201*E* − 04	0.02100
Cd	7	2.667*E* − 04 ± 2.057*E* − 05	1.143*E* − 04 ± 8.817*E* − 06	0.0001267

Open well				
Fe	8	0.05228 ± 0.03212	0.0224 ± 0.01377	0.7155
Cu	7	0.00041 ± 0.000059	0.000176 ± 0.0000253	0.006336
Zn	8	0.000356 ± 0.0000575	0.000153 ± 0.0000246	0.009231
Pb	6	0.001889 ± 0.000362	0.00081 ± 0.000155	0.02975
Cd	8	0.000275 ± 0.0000197	0.000118 ± 0.00000844	0.00003289

Spring water				
Fe	2	0.0007 ± 0.0003	0.0003 ± 0.000129	0.3918
Cu	7	0.000505 ± 0.000253	0.000216 ± 0.000108	0.3244
Zn	10	0.001917 ± 0.001086	0.000822 ± 0.000465	0.3718
Pb	9	0.001259 ± 0.000234	0.00054 ± 0.0001	0.01678
Cd	10	0.000267 ± 1.4*E* − 05	0.000114 ± 6.04*E* − 06	0.0000003236

Tap water				
Fe	6	0.01521 ± 0.01146	0.006519 ± 0.004912	0.5090
Cu	7	0.003581 ± 0.001917	0.001535 ± 0.000822	0.3549
Zn	8	0.02978 ± 0.02005	0.01276 ± 0.008594	0.4544
Pb	6	0.001556 ± 0.000222	0.000667 ± 9.52*E* − 05	0.008360
Cd	8	0.000283 ± 2.09*E* − 05	0.000121 ± 8.95*E* − 06	0.00004217

**Table 4:** Noncancer effects associated with heavy metals in drinking water in Southwestern Uganda.

Heavy metals in drinking water	Number of values	Children	Adults	*P* values
		Mean ± SEM THQ		
Borehole				
Fe	7	0.04876 ± 0.02704	0.0209 ± 0.01159	0.3708
Cu	7	0.02643 ± 0.009639	0.01133 ± 0.004131	0.1872
Zn	7	0.003222 ± 0.000386	0.001381 ± 0.000166	0.002259
Pb	7	0.4048 ± 0.1082	0.1735 ± 0.04639	0.08453
Cd	8	0.2476 ± 0.02804	0.1061 ± 0.01202	0.001598
∑THQ = HI	36	0.730812 ± 0.173305	0.313211 ± 0.074297	—

Bottled water				
Fe	1	0.002762	0.001184	NC
Cu	6	0.008056 ± 0.002412	0.003453 ± 0.001034	0.1243
Zn	7	0.0145 ± 0.0102	0.006212 ± 0.004361	0.4754
Pb	7	0.381 ± 0.0701	0.1633 ± 0.03004	0.02097
Cd	7	0.2667 ± 0.02057	0.1143 ± 0.008817	0.0001267
∑THQ = HI	28	0.6730 ± 0.1032	0.2884 ± 0.04425	—

Open well water				
Fe	8	0.07468 ± 0.04589	0.03201 ± 0.0197	0.4137
Cu	7	0.01024 ± 0.001474	0.004388 ± 0.000632	0.006336
Zn	8	0.001186 ± 0.000191	0.000508 ± 8.2*E* − 05	0.009231
Pb	6	0.4722 ± 0.09044	0.2024 ± 0.03876	0.02975
Cd	8	0.275 ± 0.01967	0.1179 ± 0.00843	3.289*E* − 05
∑THQ = HI	37	0.8333 ± 0.157665	0.35721 ± 0.06757	—

Spring water				
Fe	2	0.001 ± 0.000429	0.000429 ± 0.000184	0.3918
Cu	7	0.01262 ± 0.006319	0.005408 ± 0.002708	0.3244
Zn	10	0.006391 ± 0.00362	0.002739 ± 0.001552	0.37180
Pb	9	0.3148 ± 0.05856	0.1349 ± 0.0251	0.01678
Cd	10	0.2667 ± 0.01405	0.1143 ± 0.006023	3.236*E* − 07
∑THQ = HI	38	0.6015 ± 0.0830	0.2578 ± 0.03557	—

Tap water				
Fe	6	0.02173 ± 0.01637	0.009313 ± 0.007018	0.5090
Cu	7	0.08952 ± 0.04794	0.03837 ± 0.02054	0.3549
Zn	8	0.09928 ± 0.06684	0.04255 ± 0.02865	0.4544
Pb	6	0.3889 ± 0.05556	0.1667 ± 0.02381	0.008360
Cd	8	0.2833 ± 0.02089	0.1214 ± 0.008954	4.217*E* − 05
∑THQ = HI	39	0.8827 ± 0.2076	0.3783 ± 0.0890	—

**Table 5:** Incremental lifetime cancer risk amongst Ugandan children and adults consuming drinking water from different sources.

Cd in drinking water	Number of values	Children	Adults	*P* values
		Mean ± SEM (×10^−4^)		
Borehole	7	0.573 ± 0.0649^b^	2.87 ± 0.324^a^	0.0003196
Bottled water	7	0.617 ± 0.0476^b^	3.086 ± 0.2381^a^	3.213*E* − 05
Open well	8	0.636 ± 0.0455^b^	0.505 ± 0.0361^a^	6.557*E* − 06
Spring water	10	0.617 ± 0.0325^b^	3.09 ± 0.163^a^	4.019*E* − 06
Tap water	8	0.656 ± 0.0484^b^	3.28 ± 0.242^a^	8.152*E* − 06

Different superscripts indicate ILCR comparisons against US EPA limits. ^a^Threat of cancer; ^b^no threat.

**Table 6:** Recommended limits for selected inorganic pollutants in drinking water.

Monitoring body	Fe	Pb	Zn	Cd	Cu	Cr
	Limits in drinking water ppm					
UNBS (2014)	0.3	0.01	5	0.003	1.0	0.05
US-EPA (2009)	0.3	0.015	5	0.005	1.0	0.1
EU (1998)	0.2	0.01	3	0.005	2.0	0.05
WHO (2008)	0.3	0.01	3	0.003	2.0	0.05

## Data Availability

Data files can be accessed at https://figshare.com/s/4b2da912a42de601e165.
